# The impact of COVID-19 on service delivery systems: evidence from a survey of United States refugee resettlement agencies

**DOI:** 10.1186/s12913-022-07909-3

**Published:** 2022-04-22

**Authors:** Imelda K. Moise, Lola R. Ortiz-Whittingham, Vincent Omachonu, Ira M. Sheskin, Roshni Patel, Julia Ayumi Schmidt Meguro, Alexia Georgina Lucas, William Bice, Leila Mae Thompson

**Affiliations:** 1 Department of Geography & Sustainable Development, College of Arts & Sciences, University of Miami, 1300 Campo Sano Avenue, Coral Gables, FL 33124 USA; 2 Department of Industrial and Systems Engineering, College of Engineering, University of Miami, 1251 Memorial Drive, Coral Gables, FL 33146 USA

**Keywords:** Immigration, Migrants, Inadequate staffing, Budget cuts, Funding, COVID-19

## Abstract

**Background:**

Key to the US refugee resettlement effort is the role of non-governmental organizations (NGOs) who receive, place, and provide transitional programs and referrals to new and recently resettled refugees. Yet only one rapid assessment study thus far examined the impact of COVID-19 on service delivery systems of US refugee resettlement agencies. This exploratory study describes the capability and preparedness of US refugee resettlement agencies to provide services and care to clients during the COVID-19 pandemic.

**Methods:**

Using both telephone interviews and an internet survey, we assessed the impact of COVID-19 on service delivery, agency capacity, and preparedness of 101 US refugee resettlement agencies. Descriptive statistics were used to describe the dataset, while chi-square (χ^2^) tests were used to examine relationships by resettlement agency size (number of employees in each agency).

**Results:**

Despite a temporary pause on refugee admissions, restrictive stay-at-home orders, and refugee travel restrictions, the majority of responding US refugee resettlement agencies continued to provide specialized services and care to resettled refugees and other immigrants. Among the more important findings was that agencies that continued to provide refugee services and care onsite in their existing facilities or office rather than moving such services offsite differed by agency size [χ^2^ (9.494, *n* = 101), *p* < 0.05]. Almost all agencies (93.1%) strongly agreed or agreed that staff have timely access to COVID-19 information. Most of the refugee services were provided offsite (*n* = 72 agencies, some with multiple offices across the US).

**Conclusions:**

US refugee resettlement agencies continued to perform admirably despite a lack of funding. Future research is underway to obtain a more balanced understanding of the impact of COVID-19 on practice or operations.

**Supplementary Information:**

The online version contains supplementary material available at 10.1186/s12913-022-07909-3.

## Background

Key to the US refugee resettlement effort is the role of non-governmental organizations (NGOs) [1] who receive, place, and provide transitional programs and referrals to new and recently resettled refugees. These agencies operate under the US Department of State’s standard cooperative agreement which, in turn, specifies the services that must be provided, such as housing, employment services, and assistance in applying for social security. However, the stay-at-home order necessitated by the pandemic affected the delivery of and access to healthcare not only globally [2–4], but also across the US (beginning March 21, 2020). In US healthcare, for example [5], the most affected service delivery systems include those that focus on children’s welfare [6], psychiatric care [7], community outreach and behavioral health [8–10], and arbovirus surveillance programs [11]. In Ohio, COVID-19 caused a disruption of health and social services systems, and a decrease in volunteer availability for volunteer-dependent agencies [12]. Elsewhere, clinicians have reported challenges with the provision of services and care to refugees [13], while some agencies permanently or temporarily closed their doors [14]. Combined, these findings suggest a need to assess and monitor the impact of COVID-19 on US service delivery systems.

Aspects of service delivery affected by COVID-19 include environmental factors (e.g., work conditions), direct factors (e.g., workforce absenteeism, stress, and quarantines), and indirect factors (e.g., changes to how services are delivered and increased healthcare costs) [12, 15, 16]. For refugee resettlement agencies, service delivery systems were not only affected by the stay-at-home order but also by the Trump administration’s limit on the number of refugee arrivals, and the United Nations High Commissioner for Refugees (UNHCR)‘s suspension of refugee resettlement departures due to COVID-19. Collectively, these factors had an impact on many agencies who, in part, rely on federal funding; such funding is disbursed only when refugees arrive [17]. This has raised questions as to how social service programs should be managed during health crises or natural disasters [18]. So far, however, with the exception of a recent rapid assessment [19], the impacts of COVID-19 on US refugee services provision remain understudied in the US.

This exploratory study describes the capability and preparedness of US refugee resettlement agencies to provide services to recently resettled clients during the COVID-19 pandemic. This study was completed when the most restrictive stay-at-home orders were in place [20]. The findings below shed light on the needs of refugee resettlement programs and will help to inform needed program and policy changes that support the delivery of high-quality services to refugees during the current COVID-19 pandemic and during future health crises such as natural disasters or disease outbreaks.

## Methods

### Study design

This cross-sectional study used both telephone interviews and an internet survey to examine the capabilities and preparedness of US refugee resettlement agencies to provide services to clients during the COVID-19 pandemic. Respondents were given a choice concerning the mode by which they wished to complete the study.

### Participating agencies

Agencies of interest included those providing services and care to refugees as they arrive in the US and those responsible for placing refugees with one of their local affiliates. We found 280 resettlement agencies nationwide through internet searches. The information from these searches was verified with each respondent. Data collection occurred in May–July 2020, and targeted agency representatives (e.g., directors, executives, and any staff who was familiar with the agency’s operations). The survey was conducted at a time when the most restrictive stay-at-home orders were in effect in many states, and some states were beginning phased re-opening [20]. Trained undergraduate students in the Department of Geography Immigrant and Refugee Health course at the University of Miami conducted the telephone surveys.

### Measures

The questionnaire consisted of 31 questions divided into three sections: organizational characteristics questions (11 questions), COVID-19 and staff capacity questions (11 questions) and demographics (9 questions). Questions were multiple choice, categorical, dichotomous, open-ended, and Likert-type questions with five-point rating scales. Surveyed agencies included religious organizations, non-governmental agencies, and cultural community groups. Excluded were government facilities (e.g., detention centers and US Immigration and Customs Enforcement (ICE) facilities) because they were unable to complete a survey of this nature without time-consuming approvals.

To increase the response rate and because the study was conducted at a time when most states had stay-at-home orders, multiple contacts were made (e.g., telephone, email, Facebook, Instagram, and organization websites). Reminder notices were sent on various days of the week and at different times of the day to increase the likelihood of reaching a prospective survey respondent. Those who preferred to complete the survey online were emailed a link to the online version of the survey. The survey closed on July 10, 2020.

### Analysis

Descriptive statistical analysis was performed using IBM SPSS Statistics, version 26 [21]. Ninety-five percent confidence intervals (CIs) were developed for each percentage. Chi-square (χ^2^) tests were used to examine relationships by resettlement agency size based on the number of employees - microenterprise, 0–9; small enterprise, 10–49, medium enterprise, 50–249, and large enterprise, 250 or more). Respondents were asked to have their organizational chart with them as they respondent to the survey. Also examined were the resettlement agencies’ staff capacity and preparedness at a time when the most restrictive stay-at-home orders were in effect. Fifteen agencies did not respond to the organization type question.

## Results

**Fig. 1:**
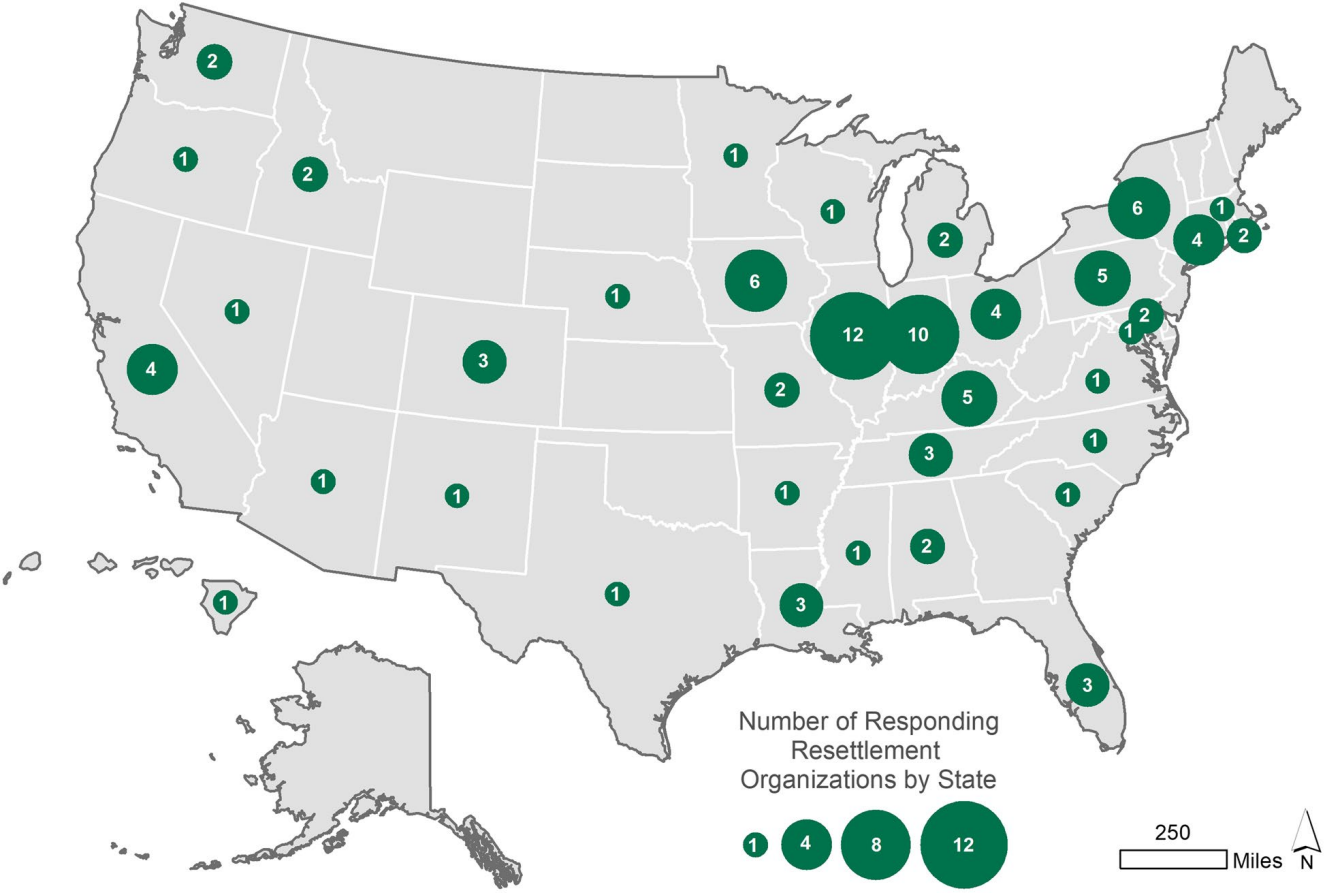
Spatial locality of responding refugee resettlemtn agencies, May–July 2020, USA

The final sample included 280 agencies. Organizations that worked with legal services and social services rather than resettlement services were excluded. Also omitted were programs that had closed permanently, and ICE detention facilities centers and jails due to non-response caused by legal restrictions. Excluded from the analysis were organizations that housed or detained refugees (*n* = 14) and questionnaires that lacked responses to critical questions (*n* = 11). This resulted in a final usable sample size of 101 (Fig. 1). Of the refugee resettlement agencies recruited for the study, 126 completed the survey (a 46.1% *response rate*). In addition, 8 respondents had incomplete responses to survey questions, 15 declined to complete the survey, and 128 could not be reached. The *cooperation rate* was 85%. That is, of the respondents that were reached, 85% completed the survey.

Table 1 presents the main characteristics of the responding refugee resettlement agencies. An important finding is that 95.1% of refugee resettlement agencies reported continuing to provide services either onsite (71%) or offsite (24%) to clients at a time when the most restrictive stay-at-home orders were in place. Only 5% discontinued services, but we suspect that many of our non-responding agencies had either closed temporarily or permanently. Closer inspection of the table shows that almost all agencies (98.8%) had guidelines in place for preventing the spread of COVID-19, while 15 agencies reported 1–4 employees as having missed work in the preceding 30 days. Over half (57.0%) of the agencies were privately run (for profit or not for profit), 33.7% were faith-based (with a religious organization, and only 9.3% were publicly run (federally or state run).

**Table 1:** Characteristics of responding refugee resettlement agencies (*n* = 101), US, 2020

Characteristic	n	%	95% CI
**Does your organization house or detain immigrants or refugees?**			
Yes	0	0	
No	101	100	
**What is your organization’s type?**			
Public, federally or state run	8	9.3	3.5–16.0
Private, for profit or not for profit	49	57.0	46.0–67.5
Faith-based, with a religious organization	29	33.7	24.1–45.1
**How long has your organization been in operation?**			
0–9 years	11	11.8	5.4–19.1
10–19 years	12	12.9	6.6–19.8
20 or more years	69	74.2	64.9–82.7
Don’t know	1	1.1	0.0–3.3
**All or most of our staff are:**			
Working on site, continuing services	24	23.8	15.8–32.7
Working off site, continuing services	72	71.3	62.4–80.2
Discontinued services	5	5.0	1.0–9.9
**How many refugees and immigrants do serve annually?**			
Less than 100 clients	10	14.1	5.6–22.5
100–499 clients	23	32.4	21.2–43.7
500–999 clients	18	25.4	15.5–35.2
1000 or more clients	20	28.2	18.3–38.0
**Do you have guidelines for preventing the spread of COVID-19?**			
Yes	82	98.8	96.3–100.0
No	1	1.2	0.0–3.7

Frequencies include refugee resettlement agencies who responded to each question. Agencies that did not respond to the question of interest (missing values) are not included

Table 2 shows that refugee resettlement agencies are split evenly when asked whether staffing levels are adequate to assist clients with COVID-19: 33.3% “strongly agree” or “agree”, 33.3% “strongly disagree” or “disagree”, and 33.3% indicated that the question does not apply. In contrast, 56.9% (*n* = 41) “strongly agree” or “agree” that staff are appropriately trained, 78.9% “strongly agree” or “agree” that their agency is practicing social distancing within their offices (*n* = 56), and 93.1% “strongly agree” or “agree” that staff are capable of interpreting guidelines issued by the CDC (*n* = 67). In addition, the majority “strongly agree” or “agree” that staff have timely access to COVID-19 information (93.1%, *n* = 67) and that their supervisor “believes that CDC guidelines are critical for mitigating COVID-19 in their agency” (84.5%, *n* = 60).

**Table 2:** Staff capacity and preparedness among refugee resettlement agencies during the COVID-19 outbreak, United States

Indicate your level of agreement about each of the following statements (*n* = 101)	Strongly agree or agree n (%)	Strongly disagree or disagree n (%)	Does not apply n (%)
Staff are capable of testing clients for COVID-19	4 (5.6)	18 (25.4)	49 (69.0)
Many staff are still hesitant to test clients for COVID-19	9 (12.7)	8 (11.3)	54 (76.1)
Staffing levels are adequate to assist clients with COVID-19	24 (33.3)	24 (33.3)	24 (33.3)
Staff are appropriately trained	41 (56.9)	13 (18.1)	18 (25.0)
Staff have timely access to COVID-19 information	67 (93.1)	3 (4.2)	2 (2.8)
Staff are capable of interpreting guidelines issued by the CDC	67 (93.1)	2 (2.8)	3 (4.2)
I am willing to work with clients with COVID-19	39 (54.9)	17 (23.9)	15 (21.1)
There is widespread support from the staff for working with clients with COVID-19	33 (45.8)	21 (29.2)	18 (25.0)
Our agency has isolation procedures in place should staff or clients show symptoms of COVID-19	42 (57.5)	7 (9.6)	24 (32.9)
Our agency has the right equipment to care for clients with COVID-19	3 (4.2)	16 (22.2)	53 (73.6)
The sanitary conditions in building are excellent	49 (66.2)	11 (14.9)	14 (18.9)
Our agency is well prepared to test clients for COVID-19	2 (2.8)	12 (16.7)	58 (80.6)
We are all expected to monitor for symptoms of COVID-19 in clients who come in for services	35 (49.3)	8 (11.3)	28 (39.4)
My agency is practicing social distancing within our offices	56 (78.9)	3 (4.2)	12 (16.9)
My agency is practicing social distancing within common areas	57 (80.3)	2 (2.8)	12 (16.9)
My supervisor believes that the CDC guidelines are critical for mitigating COVID-19 in our agency	60 (84.5)	2 (2.8)	9 (12.7)

Frequencies include refugee resettlement agencies who responded to each question. Agencies that did not respond to the question of interest (missing values) are not included

Table 3 shows wide variation in service provision mode based on refugee resettlement agency size, type, and length in operation (Table 3). Small (*n* = 22, 72.3%) to medium (*n* = 22, 95.7%) enterprises were more likely to continue providing services to client offsite as well as onsite. Of those that continued to provide services offsite, most were faith-based organizations, and most had been in operation for more than 20 years.

**Table 3:** Comparing service provision mode by refugee resettlement agency size, type and length in operation

	Working on site, continuing services n (%)	Working off site, continuing services n (%)	χ^**2**^	***P-***value
**Organization size**				
Microenterprise, 0–9 employees	10 (43.5)	13 (56.5)	9.494	0.023*
Small enterprise, 10–49 employees	10 (31.3)	22 (68.8)		
Medium enterprise, 50–249	1 (4.3)	22 (95.7)		
Large enterprise, 250 or more	1 (25.0)	3 (75.0)		
**Operation type**				
Public, federally or state run	2 (25.0)	6 (75.0)	1.897	0.387
Private, for profit or not for profit	10 (20.4)	39 (79.6)		
Faith-based, with a religious organization	10 (34.5)	19 (65.5)		
**Time in operation**				
0–9 years	5 (45.5)	6 (54.5)	6.545	0.088
10–19 years	4 (33.3)	8 (66.7)		
20 or more years	14 (20.3)	55 (79.7)		
Don’t know	1 (100.0)	0 (0.0)		

Microenterprises are defined as having 0–9 employees, small enterprises as having 10–49 employees, medium enterprises as having 50–249 employees, and large enterprises as having 250 or more employees. Agencies that did not respond to the question of interest (missing values) are not included. Bivariate analyses using χ2 test was used, with *p < 0.05

## Discussion

This paper offers a unique exploration of the impact of COVID-19 on service delivery systems experienced by US refugee resettlement agencies during the early months of the COVID-19 pandemic. Understanding this impact is vital, as these agencies are typically the first agencies to engage with refugees upon arrival, and can support refugees who, like minority populations, may be at higher risk for COVID-19 [22]. In addition, in the early resettlement period, refugees encounter multiple potential stressors and competing priorities (e.g., adjusting to a new country, finding employment, learning a new language, and navigating complex, unfamiliar healthcare systems) [23]. Our findings, collected via cross-sectional analysis, facilitated this understanding as the impact of COVID-19 on service delivery systems were identified directly by agencies experiencing and working to address them.

While refugee resettlement agencies are split almost evenly on whether staffing levels are adequate to assist clients with COVID-19, most continued providing services onsite (*n* = 24 agencies) or offsite (*n* = 72 agencies), and most had multiple offices. Most agencies also had staff that missed work, tested positive for COVID-19, or quarantined in the preceding 30 days, even though the numbers of the affected staff varied by refugee resettlement agency size, type, and length in operation. Microenterprise agencies (with 0–9 employees), were disproportionately affected compared to larger agencies. Our findings demonstrates that despite the existence of refugee policies aimed at curbing resettlement and travel restrictions, refugee resettlement agencies had to remain operational to continue assisting recently resettled refugees and to resettle refugees that the US continued to accept during the pandemic. For example, the US admitted 1584 refugees in February 2020 and 1110 refugees in March 2020, with admission suspended on March 17 when the United Nations High Commissioner for Refugees (UNHCR) suspended resettlement departures due to the growing COVID-19 pandemic [24].

The capabilities of US refugee resettlement agencies may be attributed to these agencies’ preparedness and response to the COVID-19 pandemic. For example, at the time of this study during the early stages of the COVID-19 pandemic, nearly 56.9% (*n* = 41) of refugee resettlement agencies “strongly agree” or “agree” that staff are appropriately trained, 78.9% “strongly agree” or “agree” that their agency is practicing social distancing within their offices (*n* = 56), and 93.1% “strongly agree” or “agree” that staff are capable of interpreting guidelines issued by the CDC (*n* = 67). In addition, the majority “strongly agree” or “agree” that staff have timely access to COVID-19 information (93.1%, *n* = 67) and that their supervisor believes that the CDC guidelines are critical for mitigating COVID-19 in their agency (84.5%, *n* = 60). Combined, these institutional aspects reflect agency readiness [25, 26]. However, the observed split in views on staffing levels raise concerns about the ability of refugee resettlement agencies to respond in a timely and effective manner to the needs of resettled refugees during health crises.

### Strengths and limitations

An important strength of this study is the inclusion of US refugee resettlement agencies with varying organization type (public, private, and faith-based), years in operation, caseloads, capacities, and regional representation. This contributed to in-depth insight into the perspectives of refugee resettlement agencies’ capacity and preparedness to serve clients during COVID-19 and allowed for comparisons of service provision capabilities by agency size, type, and length in operation.

Not assessing the impact of COVID-19 on the lives of resettled refugees is a limitation, as the opinions of refugees might differ from that of the respondents at the refugee resettlement agency. Since the study was conducted during a period when the country was under strict stay-at-home orders, it was not possible to compare pre- and post-COVID-19 impacts on refugee resettlement agencies, and this may have led us to miss some agencies. This is an important issue for future research. Likewise the US Census Bureau [27] has recently developed and implemented an alternative nonresponse adjustment while several lines of recent evidence also suggest ways to mitigate nonresponse rates [28–31] that can be used in future studies with good effect under similar contexts. Further, the generalizability of these results is subject to certain limitations. For instance, although we had regional representation across the US, the results presented here are not representative of some agencies that serve refugees (e.g., prisons). The small sample size for some types of agencies (public vs. public), particularly large enterprises, limited more robust statistical analysis and comparison beyond basic descriptive statistics.

Despite its exploratory nature, this study offers insight into the impact of COVID-19 on service delivery systems, particularly on refugee resettlement agencies, and might inform strategies to address service delivery system challenges during times of crisis or disasters.

### Implications for research and practice

Future research could focus on understanding pre- and post-COVID-19 impacts on service delivery systems of refugee resettlement agencies, as well as on the perspectives of resettled refugees. Understanding such perspectives is vital for insight into how such cumulative adversities experienced by the refugee community have widened the gaps to social services, and healthcare access, including social support [19]. More specifically, studies could unravel the circumstances under which agency capacities or preparedness to assist refugees varied or changed. Lastly, qualitative research such as focus groups could establish the link between survey data and perspective of agencies working with refugee populations during the post-COVID period. These methods could also help better understand refugee outcomes and experiences.

In practice, this study suggests the potential benefit of actively addressing funding sources to allow refugee resettlement agencies to continue providing specialized services to current refugees during times of crisis or when the number of arriving refugees is low. Moreover, a few models exist, such as the hybrid funding model, that can allow agencies to provide services privately without federal funding, with federal funding and/or with private reimbursement or upfront funding [32]. Notably, although UNHCR suspended resettlement departures due to COVID-19 [24], US refugee resettlement agencies continued to provide services to their clients. Findings also draw attention to the need for extra vigilance in the monitoring and assessment of refugee resettlement agencies as well as other public-serving agencies during pandemics or natural disasters that may require unanticipated levels of service consumption and delivery. Now that the Biden administration has increased the cap of refugees to 125,000 for 2022 [33], reinstating the affected infrastructure of resettlement programs may require investment in resettlement infrastructure to bring it to its former state before refugees arrive.

## Conclusion

This study explored the capability and preparedness of US refugee resettlement agencies to provide services to clients during the COVID-19 pandemic. Like our previous study [11], we observed that US refugee resettlement agencies continued to perform despite a lack of funding. This posture can be attributed to the professional predisposition of refugee resettlement agencies to helping the underserved, regardless of capacity or funding levels. Future research on US refugee resettlement agencies’ perspectives by organization type and funding is needed to obtain a more balanced understanding of their practice or operations.

## Supplementary Information

**Additional file 1.**

## Data Availability

Data are available upon request to the corresponding author.

## Abbreviations

COVID-19: Coronavirus disease 2019
US: United States
UNHCR: United Nations High Commissioner for Refugees
FY: Fiscal Year
ICE: Immigration and Customs Enforcement
IRB: Institutional Review Board
CDC: Centers for Diseases Control and Prevention
